# Quantification of phenobarbital-induced ataxia in dogs with idiopathic epilepsy

**DOI:** 10.3389/fvets.2023.1168335

**Published:** 2023-05-18

**Authors:** Tamara Sherif, Friederike Twele, Sebastian Meller, Alexandra Müller-Anders, Holger A. Volk

**Affiliations:** Department of Small Animal Medicine and Surgery, University of Veterinary Medicine Hannover, Hannover, Germany

**Keywords:** ataxia, epilepsy, dog, movement, locomotion, gait analysis

## Abstract

Ataxia is a clinical sign seen in several neurological diseases and has been reported as an adverse effect of anti-seizure medication such as phenobarbital (PB). Efforts to objectify canine ataxia in order to provide appropriate treatment or monitor adverse effects of drugs remain limited. Automated quantitative gait analysis can be useful for the acquisition of objective data for the evaluation and monitoring of ataxia in dogs. The aim of this prospective clinical study was to examine the gait characteristics of dogs with PB induced ataxia and compare them with healthy dogs using a computer- and treadmill-based gait analysis system. Six healthy dogs and five dogs with idiopathic epilepsy (IE) with PB-induced ataxia underwent video- and computer-assisted gait analysis during slow walking (maximum speed of 0.7 m/s) on a treadmill with four ground reaction force plates (one plate per limb). Kinetic and spatio-temporal gait parameters of dogs’ locomotion were analyzed, including individually calculated coefficients of variation. Dogs with IE treated with PB showed higher variability in spatio-temporal but not in kinetic gait parameters. Double support phase of gait cycles was increased on the cost of single support and swing phases. Body weight standardized ground reaction forces in vertical, craniocaudal, and mediolateral direction were severely affected by ataxia. Compensatory mechanisms in locomotion of dogs with PB-induced ataxia included spatio-temporal and kinetic gait characteristics, most likely in order to compensate imbalance caused by limb incoordination.

## 1. Introduction

Ataxia is a neurological clinical sign and is defined as “a disturbance in the coordination of movement or collaboration of muscle groups” (1). It can be accompanied by “loss of the orientation of the body axis” (2). Incoordination can be considered as the disability to proceed a cyclical relationship of temporal and spatial gait characteristics, especially between certain areas of the body and limbs (3). Abnormal gait in dogs, apart from neurological origin, can also be caused by musculoskeletal, cardiovascular, or metabolic/endocrine events (2) which make detailed examination indispensable. Ataxia can also be seen as an adverse effect of drugs, especially anti-seizure drugs (ASDs). A recent study has shown that ataxia was one of the most common noted adverse effects of phenobarbital (PB), which remains the first-choice ASD used in dogs (4). Owners, as proxy for their pet’s quality of life assessment, find ataxia and sedation the most cumbersome adverse effects, effecting their own quality of life significantly (5). Ataxia is usually subjectively assessed in most studies, but there is a need to better quantify and characterize the level of ataxia. Currently only one study has attempted to quantify ataxia in dogs with IE treated with PB using a manual gait analysis system, which was relatively simple, but very time consuming. The analysis focused mainly on stride length and lateral paw placement (6). Hence, further research is needed into measuring drug related ataxia more objectively.

Previous studies on objective gait analysis in dogs aimed at evaluating locomotion parameters of healthy dogs (7–11), dogs with orthopedic diseases (12, 13) or improvement of gait characteristics after medical or surgical treatment (14–16). Although neurologically caused changes in gait have been a target of several studies before (6, 17–23) there remains a lack of information on the potential of using treadmills to objectively assess ataxia in dogs.

A video- and computer-assisted gait analysis system can detect kinetic and spatio-temporal gait parameters of dogs’ locomotion on a treadmill. Kinetics describe the “study of forces occurring during motion” (24), mainly revealing ground reaction forces in vertical, cranio-caudal and medio-lateral direction transmitted to force plates by the dog’s moving limb. It has been used as a convenient tool in objectively evaluating musculoskeletal conditions, as well as different therapeutic options (24–26), providing limb-specific information (24).

The aim of the current study was to evaluate if the variability of temporal and spatial gait characteristics, as well as ground reaction force parameters (kinetics) of dogs with medication-induced ataxia differ significantly from those of a control group.

## 2. Materials and methods

Experiments were performed according to the EU Council Directive 210/63/EU and the German Law on Animal Protection. Ethical approval for the study was granted by an ethical committee (according to §15 of the German Animal Welfare Act) and the government agency (Lower Saxony State Office for Consumer Protection and Food Safety, LAVES) responsible for approval of animal experiments in Lower Saxony (reference number for this project: 20A555).

### 2.1. Data collection

**Figure 1:**
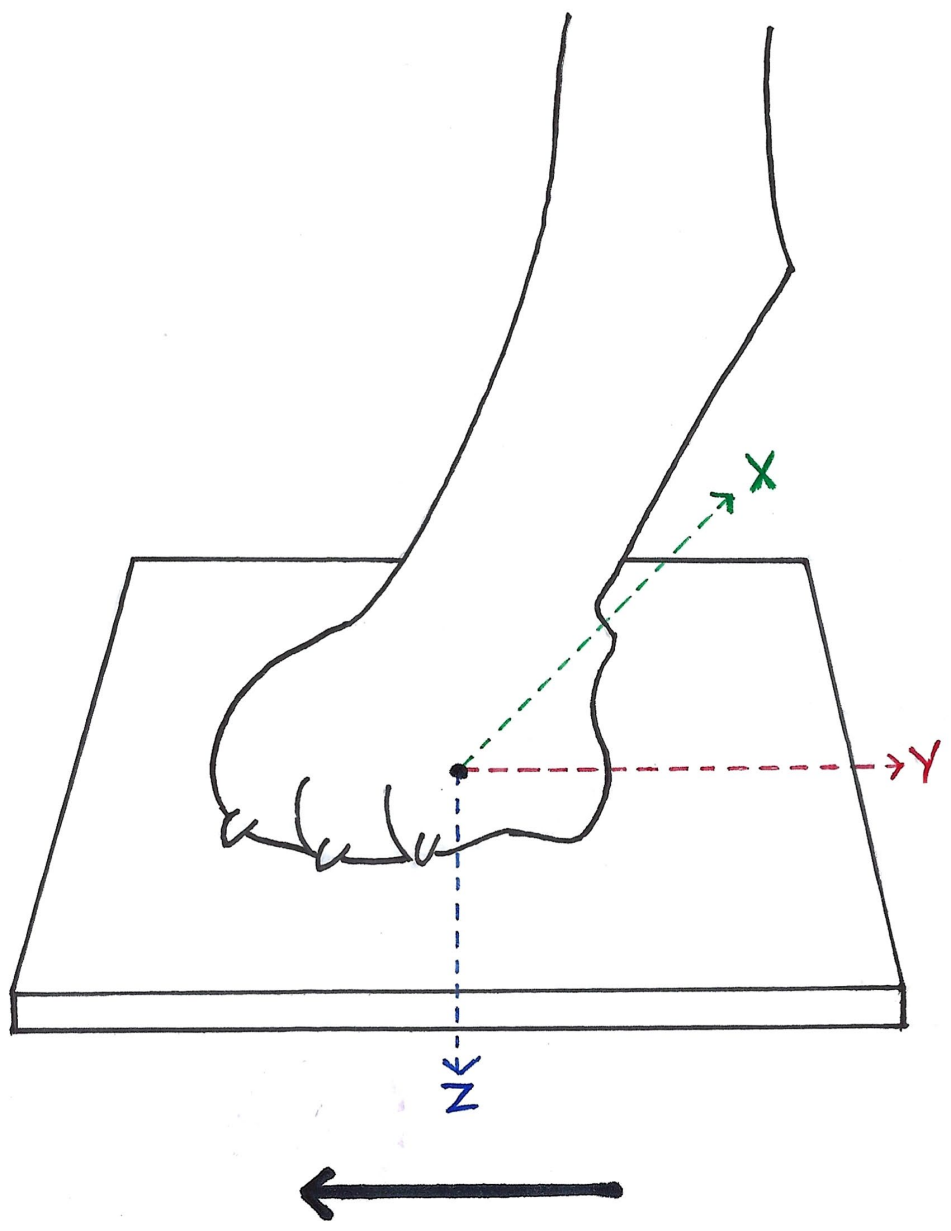
Ground reaction forces (GRF) measurable during stance phase of each paw in vertical (Fz, blue arrow), cranio-caudal (Fy, red arrow), and medio-lateral (Fx, green arrow) direction, according to McLaughlin et al. (26). Solid arrow = direction of forward progression.

Data collection was performed at the Department of Small Animal Medicine and Surgery of the University of Veterinary Medicine Hannover. In this prospective clinical study, gait parameters of healthy dogs were compared to dogs with IE treated with PB. For this study, all patients with an orthopaedically caused gait aberration were excluded. Therefore, a detailed history, as well as general, orthopedic and neurological examination were performed. General examination was necessary in order to rule out other clinical conditions such as cardiovascular insufficiency that were incompatible with participation in a treadmill based study. As most patients with ataxia also have a neurological deficit (2), a neurological examination has been conducted on all dogs participating in this study. Neurological examination can furthermore help define the exact neuroanatomical localization, such as the spinal cord, the vestibular system, or the cerebellum (2). Kinetic gait characteristic data were collected simultaneously as described in detail in previous studies in the gait analysis laboratory at the University of Veterinary Medicine Hannover (9, 11, 14, 15, 27). The gait laboratory was composed of a treadmill with four integrated force measuring plates including high-speed cameras. It allowed measuring ground reaction forces (Figure 1). The electronic four-belt treadmill (TM-07-B, Bertec Corp, United States) and an additional digital high-speed video camera (pilot piA640-210gc; Basler, Germany) recorded the gait of participating dogs from the left-hand side lateral position. The devices listed were set and controlled using the Vicon Nexus software (ver 1.8.5; Vicon Motion Systems Ltd., United Kingdom) as well as Bertec Treadmill Control Panel software (ver 1.7.12; Bertec Corp, United States). Calibration of the motion capture system prior to use was necessary, as well as a training phase prior to recording until the dogs walked in an undisturbed manner. For the dogs to be able to walk smoothly and regularly treadmill speed was adjusted to a maximum of 0.7 m/s.

### 2.2. Data processing

**Figure 2:**
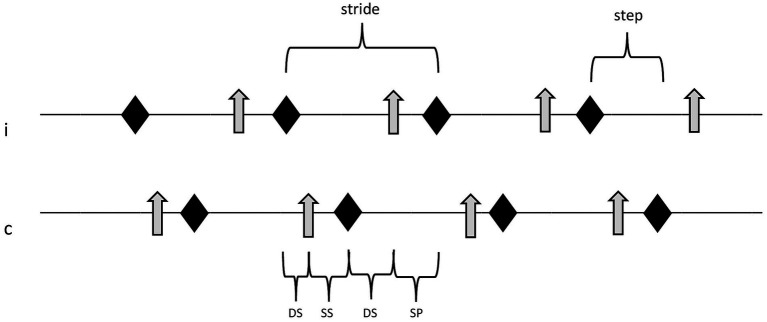
Sequence of locomotion events in one limb pair (thoracic or pelvic limbs). Double support phases, single support phase, and swing phase of the ipsilateral paw equal 100% of one full stride. rhomb, event “paw strike”; arrow, event “paw off”; i, ipsilateral paw; c, contralateral paw; DS, double support phase; SS, single support phase; and SP, swing phase.

Only the trials with regular gait and the greatest number of consecutive strides with the least amount of head turning or walking speed changes were selected for each dog for further analysis of a total of 50 complete strides (Figure 2). Data of ground reaction forces (GRF) were filtered through a low-pass 10-Hz finite-impulse response filter. Strike-down and lift-off of paws, as well as start and end of presence of measured vertical GRF were defined using the high-definition video material. All data were exported to a Microsoft Excel 2010 spreadsheet (Microsoft Corp, United States). Variables were individually evaluated for left and right thoracic and pelvic limbs. Supplementary Table 1 shows definitions of spatio-temporal and kinetic gait parameters in further detail.

All GRF data were standardized for each study participant and expressed as percentage of its body weight using the following equation:


$$\mathrm{GRF}=\mathrm{Fz};\mathrm{Fy};\mathrm{Fx}/\left(\mathrm{body}\;{\mathrm{weight}}^{*}9.81\right)$$

(9.81 = gravitational force in New/kg).

In addition, symmetry indices, relative step time, and relative step length were calculated using the following equations:


$$\mathrm{Symmetry index}\;\left(\mathrm{SI}\right)=100\%-\left[{\left(\mathrm{PFz}\;\mathrm{left}\;\mathrm{paw}/\mathrm{PFz}\;\mathrm{right}\;\mathrm{paw}\right)}^{*}100\right]$$

[modified formula by Budsberg et al. (28)].


Relative step time=step time/stride time

Relative step length=step length/stride length

Finally, the coefficient of variation (CV) of every gait parameter was calculated, utilized here as in previous studies looking at ataxia in dogs (6, 17, 29), horses (30), and humans (31) in order to enable relative comparison of interindividual measurements.


CV=standard deviation/mean

### 2.3. Statistical analysis

Statistical analysis was performed with GraphPad Prism 9 software (GraphPad Prism 9.2.0, GraphPad Software, Inc., United States). Differences in age, sex status, and body weight between the groups were tested with an unpaired *t*-test. Data were analyzed for normality of distribution with a Kolmogorov–Smirnov test and visual inspection of residual plots. After Gaussian distribution was rejected multiple Mann–Whitney test were performed. In all statistical tests a value of *p* of less than 0.05 was considered significant. *p* values were then re-evaluated using a False Discovery Rate (FDR) to reduce Type-I error due to multiple comparisons. The method chosen for setting FDR was developed by Benjamini et al. (32). Data were not-normally distributed and are therefore shown as median with range.

## 3. Results

### 3.1. Study participants/animals

Eleven dogs of various breeds, ages, sex, and body weights were included in the current study. The control group consisted of six healthy Beagle dogs [age 4 (2–4) years; body weight 15.5 (12.7–16.7) kg]. All dogs of the control group were clinic-owned and were habituated to walk on the treadmill in advance. The PB-treated group consisted of five dogs [one Australian Shepherd, one Rhodesian Ridgeback, one Giant Schnauzer, two cross breed dogs; age 5 (4–13) years; body weight 33.8 (9.7–41.4) kg] that were selected after excluding general and orthopedic diseases and had ataxia as a PB-induced adverse effect. All dogs of the PB group were diagnosed with IE confidence level tier II (4) receiving long-term PB treatment without any PB dosage adaptions for more than 1 year. However, PB dosages and severity of ataxia differed between the dogs. The differences in age and sex status between the two groups were not statistically significant. The differences in body weight between the control group and the group of PB-induced ataxia were statistically significant (*p* = 0.025) with the control group being lighter.

### 3.2. Spatio-temporal gait parameters

#### 3.2.1. Parameter values

As presented in Table 1A, post FDR re-evaluation most parameter values were severely affected. Comparing values of 50 strides showed significant differences between the groups in stride length, stride time, step length, relative step length, step time, single support phase, and double support phase of the thoracic and pelvic limbs, as well as stance time and swing phase of the thoracic limb pair. In contrast to most stride phases, no differences were seen in relative step time of both, thoracic and pelvic limbs. On the other hand, all differences found were highly significant.

**Table 1:** Statistical analysis of parameter values and coefficients of variation (^*^p ≤ 0.05, Mann–Whitney test, FDR Q = 0.05) of spatio-temporal (A) and kinetic (B) gait parameters.

Spatio-temporal	Parameter values		Coefficients of variation		Kinetic	Parameter values		Coefficients of variation	
	*p*- value	FDR threshold	*p*- value	FDR threshold		*p*- value	FDR threshold	*p*- value	FDR threshold
Stride length T	<0.0001^*^	0.0042	0.0303	0.0060	PFz T	<0.0001^*^	0.0042	>0.9999	0.05
Stride length P	<0.0001^*^	0.0050	0.1255	0.0122	PFz P	<0.0001^*^	0.0050	0.7922	0.05
Step length T	<0.0001^*^	0.0060	0.0043	0.0042	MFz T	<0.0001^*^	0.0060	0.5368	0.0200
Step length P	<0.0001^*^	0.0074	0.1255	0.0167	MFz P	<0.0001^*^	0.0074	0.0519	0.0050
Stride time T	<0.0001^*^	0.0094	0.7922	0.05	PFy T	<0.0001^*^	0.0094	0.4286	0.0139
Stride time P	<0.0001^*^	0.0122	0.9307	0.05	PFy P	<0.0001^*^	0.0122	0.4286	0.0102
Step time T	<0.0001^*^	0.0167	0.2468	0.0240	PFx T	<0.0001^*^	0.0167	0.7922	0.0313
Step time P	<0.0001^*^	0.0240	0.0823	0.0074	PFx P	<0.0001^*^	0.0240	0.1255	0.0062
Stance time T	<0.0001^*^	0.0375	0.3290	0.0375	IFz T	<0.0001^*^	0.0375	>0.9999	0.05
Stance time P	<0.0001	0.05	0.5368	0.05	IFz P	<0.0001	0.05	0.1255	0.0078
Swing time T	<0.0001	0.05	0.0823	0.0094	Range Fz T	<0.0001^*^	0.0042	0.6623	0.0240
Swing time P	<0.0001	0.05	0.0043^*^	0.0050	Range Fz P	<0.0001^*^	0.0050	0.9307	0.05
rel. Step length T	<0.0001^*^	0.0050	0.0043^*^	0.0050	STDEV Fz T	<0.0001^*^	0.0060	>0.9999	0.05
rel. Step length P	<0.0001^*^	0.0062	0.1255	0.0313	STDEV Fz P	<0.0001^*^	0.0074	0.7922	0.0375
rel. Step time T	0.7988	0.05	0.1775	0.05	Range Fy T	<0.0001^*^	0.0094	0.5368	0.0167
rel. Step time P	0.6856	0.05	0.0519	0.0200	Range Fy P	<0.0001^*^	0.0122	0.0822	0.0050
Single support T	<0.0001^*^	0.0078	0.0173^*^	0.0078	STDEV Fy T	<0.0001^*^	0.0167	0.7922	0.05
Single support P	<0.0001^*^	0.0102	0.0087^*^	0.0139	STDEV Fy P	<0.0001^*^	0.0240	0.2468	0.0122
Double support T	<0.0001^*^	0.0139	0.6623	0.05	Range Fx T	<0.0001^*^	0.0375	0.0519	0.0042
Double support P	<0.0001^*^	0.0200	0.4286	0.05	Range Fx P	<0.0001	0.05	0.1775	0.0094
Swing phase T	<0.0001^*^	0.0313	0.0043^*^	0.0062	STDEV Fx T	<0.0001	0.05	0.1255	0.0074
Swing phase P	<0.0001	0.05	0.0087^*^	0.0102	STDEV Fx P	<0.0001	0.05	0.0823	0.0060

50 strides of a non-ataxic control group as well as 50 strides of an ataxic study group were analyzed and compared. T, thoracic limbs; P, pelvic limbs; N, number of steps; SD, standard deviation; CV, coefficient of variation; highlighted cells, absolute values (restricted comparability).

#### 3.2.2. Coefficients of variation

**Figure 3:**
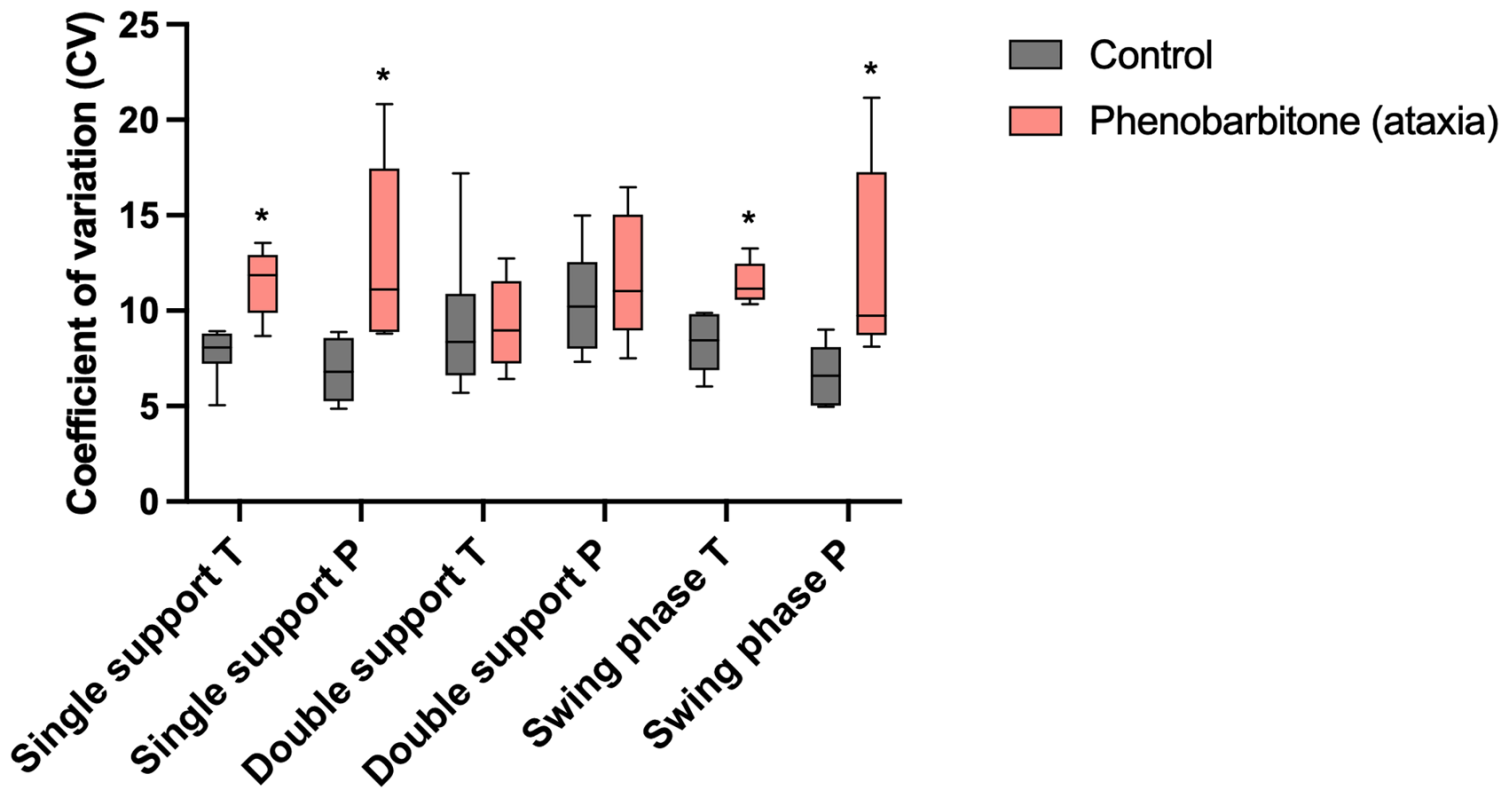
Coefficients of variation (CV) of spatio-temporal gait parameters. CVs of a non-ataxic control group were compared to those of a PB-treated ataxic IE study group. CVs of single support phase, as well as swing phase of both limb pairs showed significantly higher variability in the study group than in controls. ^*^p ≤ 0.05, Mann–Whitney test. T, thoracic limbs; P, pelvic limbs.

Table 1A shows that stride phase parameters were affected. Significant differences regarding CVs of relative step length of the thoracic limbs, swing time of the pelvic limbs and single support phase, as well as swing phase of both limb pairs were found, with PB showing higher variability. Spatio-temporal parameters that were not directly linked to stride phases showed no significant differences. Figure 3 presents differences in CVs of stride phases between groups.

### 3.3. Kinetic gait parameters

#### 3.3.1. Parameter values

Differences were detected for mean and peak GRF in vertical, craniocaudal, and mediolateral direction of the thoracic and pelvic limbs, impulse of vertical GRF of the thoracic limbs, as well as most range and standard deviation of all detected steps of both limb pairs as presented in Table 1B. All differences found were highly significant. Supplementary Table 4 shows that no significant differences between the groups were found for symmetry indices of the pelvic limbs.

#### 3.3.2. Coefficients of variation

As presented in Table 1B, no differences were found between the groups comparing CVs of kinetic gait parameters.

## 4. Discussion

In this study, objective gait analysis in dogs with IE treated with PB (4, 6) revealed differences in gait parameters and their CVs compared to a healthy control group. Objective gait analysis on a treadmill with four built-in ground reaction force plates showed increased spatio-temporal variability, a shift in dispersion of stride phases and severe alterations in ground reaction forces. This study showed that ataxia secondary to PB treatment can be objectively quantified with a video- and computer-assisted gait analysis system.

Similar to other gait studies into ataxia (6, 17, 29–31), CV was used in the current study to null the effect of size or weight influences. CV is a descriptive parameter used to quantify the margin of data, in this case the intraindividual variability of a gait parameter and thus allows relative comparison of interindividual measurements. The higher the CV, the more variable is the data. In 2008, Hamilton stated that using CV in studies with ataxic patients sets a focus on coordination consistency and can thus be convincingly compared to the variability of another patient’s individual gait characteristics (29). This approach was also applied successfully in canines (6, 17) and horses (18). CVs of some but not all spatio-temporal gait parameters were significantly higher in PB-treated dogs with IE. Contrary to expectations, CVs of ground reaction force parameters (kinetics) were not affected by PB-induced ataxia, indicating that variability of the ataxic gait does not strike kinetics at the same expense as spatio-temporal gait parameters. Still, a tendency of higher variability in ataxic dogs could be seen. An increase in variation of spatio-temporal gait characteristics, as illustrated in the current study, was found in previous studies in ataxic dogs (6, 17) and humans (20, 33–35) and can be interpreted as primitive mechanisms of stabilizing movement. Current scientific considerations on how quadrupeds stay in strict control of their coordination and balance, lead to the uncontrolled manifold theory, that an individual’s head and limbs are targeting at keeping its center of mass (CoM) stable by variation in motion of the limbs (36).

Differences in absolute values can be explained in parts by differences in limb sizes of participating dogs. Dogs with shorter legs take smaller steps independent of their status in gait coordination ability (7). For better comparability, relative values are more valid indicators. In this study, the majority of values were set in relation to the individual’s body weight or stride cycle or were illustrated as indices. For all gait parameter values, data from 50 steps of each participant’s ipsilateral paw (100 steps per individual limb pair in total) were compared instead of simply comparing 1 mean per individual and parameter.

The changes in stride phases could potentially be explained by the animal aiming at distributing body weight to a higher number of limbs for a sustained time to improve stability in gait. This matches results of a study by Stolze and colleagues, in which typical features of cerebellar ataxic gait in humans have been investigated (20). The study showed extended stance and a double support time in humans with cerebellar diseases (20). The cerebellum is responsible for coordinating locomotion and motoric fine-tuning (37). The gait alterations in dog with IE treated with PB is most likely therefore related to cerebellar ataxia. In the current study, differences in ground reaction forces were seen between groups. To improve comparability between groups the ground reaction forces were normalized to body weight as formerly described. Major differences in GRF could be detected, indicating that GRF might also contribute to implementing the uncontrolled manifold theory and ultimately keeping CoM stable. Although, analyzing of relative data can help compare between different breeds, the impact of a rather uniform control group being compared to an experimental group of different dog breeds, remains unclear. Previous studies found that the physiological relation of vertical GRF (Fz) between thoracic and pelvic limb pairs depended on the dog’s individual body size (38). This indicates that different dog breeds seem to have slightly different gait characteristics. In order to validate the results found in this study and to exclude possible influences on differences in stride phases or GRF, further studies with uniform study groups are needed.

Albeit, the potential of treadmills used to objectively assess ataxia in IE dogs under PB treatment was not evaluated before, neurologically caused changes in gait have already been investigated in previous studies. In 2016, Suiter and colleagues included IE dogs under different ASD treatments and mainly focused on six spatio-temporal parameters (6). They manually analyzed their data, making this setup not only limited in the number of parameters detectable but also expensive in time and effort. In a study by Olsen and colleagues spatio-temporal gait parameters in dogs with Chiari-like malformation and syringomyelia were evaluated (17). Data acquisition was performed on a grid mat and video and image analysis software was used for analysis. Similar to our study, CV was calculated to quantify gait variability, although kinetic parameters were not included. In 2007, Hamilton and colleagues used a digital motion capture system to measure forelimb-hindlimb coordination in dogs with spinal cord injury (21). The focus was set on the use of quadrupeds as a model population for human spinal cord injury and the functional effect of therapeutic interventions. For gait analysis, this study describes the use of motion capture and analysis of one single parameter. Olby et al. (23) performed a treadmill based study scoring gait in dogs with thoracolumbar spinal cord injury. They used a one-belt treadmill and manually analyzed stepping and coordination scores.

Phenobarbital is considered first-line treatment in canines with IE, and ataxia is a frequently observed adverse effect (4, 6). PB is a medication of the barbiturate type with affinity to gamma-aminobutyric acid_A-_ (GABA_A_) receptors and increases the probability that in presence of the neurotransmitter GABA corresponding channels open which leads to more frequent hyperpolarization of the inhibitory synapse due to influx of chloride ions (39–41). This chemical bond simultaneously effects GABA_A_ receptors of the entire central nervous system (CNS) including the cerebellum, which can ultimately lead to cerebellar dysfunction and successional changes in locomotion (20). This needs to be considered when comparing the results to studies of spinal cord injury as these dogs also have paresis in addition to ataxia, which can influence the gait further. Higher variability in spatio-temporal gait parameters was found in one previous study with manual analysis of 50 strides of dogs with IE chronically treated with PB with higher variability (CVs) in stance time and lateral paw placement of both limb pairs which matches our results (6). In dogs with IE treated with the ASD imepitoin, this effect was not detected (6). PB dosage or the period around the beginning of PB treatment both affect severity of ataxia in dogs with IE. Further studies in dogs with equivalent PB serum levels, PB dosages or durations of PB treatment are therefore needed to study the impact of PB on canine ataxia in greater detail.

## 5. Study limitations

This study was approved by Lower Saxony State Office for Consumer Protection and Food Safety in Oldenburg, Germany. The number of participating dogs in this study was therefore smaller than originally wished for, limiting the overall number of dogs in both groups. For the control group, we were furthermore limited to use clinic-owned Beagle dogs, reducing diversity of breeds remarkably. This also limited us to match the control group for age, size, and weight. A further limitation was that we had not used PB in healthy dogs, so that we cannot rule out completely that IE could have caused gait alterations. Another limitation was that during kinetic gait analysis, stride events “paw strike” and “paw off” were set mainly according to start and end of detection of vertical ground reaction forces (Fz). Hence results for craniocaudal (Fy) and mediolateral (Fx) GRF might be less valid than for Fz. The authors suggest that further studies including kinematic analysis and electromyographic activity detection as performed in previous publications (42) with larger sample sizes and higher diversity of participating dog breeds may be helpful for further specification of canine ataxic gait. In addition, study groups with different neuroanatomical localizations causing ataxia could help to characterize the different ataxia forms better.

## 6. Conclusion

To the best of authors’ knowledge, this is the first study aiming at objectively measuring medication-induced ataxic gait parameters in canines in comparison to a non-ataxic control group using a four-force plate treadmill system. Objective gait analysis with high sensitivity, as used in this study potentially helps neurologist to detect adverse effects of PB treatment early and more reliably than by subjective observation. This consecutively might help adapting ASD dosage of IE patients in order to maintain their quality of life, as well as possible and ultimately optimize IE treatment monitoring. Even though gait analysis on a treadmill was not invasive nor distressful for participating dogs, the expenditure of time and work for data collection and analysis was proportionally high. Body-worn sensors and machine learning techniques might be a more practical alternative for routine clinical use, even though the number of gait parameters measured could be limited (43).

## Data availability statement

The raw data supporting the conclusions of this article will be made available by the authors, without undue reservation.

## Ethics statement

The animal study was reviewed and approved by Lower Saxony State Office for Consumer Protection and Food Safety, LAVES (reference number for this project: 20A555). Written informed consent was obtained from the owners for the participation of their animals in this study.

## Author contributions

TS, FT, SM, and HV performed patient acquisition and planned the experiment(s). TS and AM-A ran the experiment. TS analyzed the data. TS, FT, SM, AM-A, and HV participated in manuscript writing. All authors contributed to the article and approved the submitted version.

## Funding

This Open Access publication was funded by the Deutsche Forschungsgemeinschaft (DFG, German Research Foundation)—491094227 “Open Access Publication Funding” and the University of Veterinary Medicine Hannover, Foundation.

## Conflict of interest

The authors declare that the research was conducted in the absence of any commercial or financial relationships that could be construed as a potential conflict of interest.

## Publisher’s note

All claims expressed in this article are solely those of the authors and do not necessarily represent those of their affiliated organizations, or those of the publisher, the editors and the reviewers. Any product that may be evaluated in this article, or claim that may be made by its manufacturer, is not guaranteed or endorsed by the publisher.
