# Supplementary material for: Quantification of phenobarbital-induced ataxia in dogs with idiopathic epilepsy
Source: Front Vet Sci. 2023 May 18;10:1168335. doi: 10.3389/fvets.2023.1168335 (PMC10232958; doi:10.3389/fvets.2023.1168335)
Supplement: Supplementary file 1 [file Data_Sheet_1.PDF]

Supplementary Material

Quantification of phenobarbital-induced ataxia in dogs with idiopathic epilepsy

Tamara Sherif[1], Friederike Twele[1], Sebastian Meller[1], Alexandra Müller-Anders[1], Holger A. Volk[1*]

[1] Department of Small Animal Medicine and Surgery, University of Veterinary Medicine Hannover, D-30559 Hannover, Germany

*** Correspondence:**
Holger A. Volk
holger.volk@tiho-hannover.de

1 Supplementary Tables

Supplementary Table 1. Definitions of gait parameters measured or calculated on behalf of this study. T = thoracic limb, P = pelvic limb, walking speed = stride length/stride time

**Parameter**	**Unit**	**Definition**	**Further Information**
**Spatio-temporal**			
Stride length	mm	Distance between paw marker position of ipsilateral limb during one stride.	Treadmill belt length that passed during full gait cycle
Stride time	s	Time between successive ipsilateral paw strikes.	Full gait cycle
Step length	mm	Distance between paw marker position of ipsilateral limb during one step.	
Step time	s	Time between contralateral and successive ipsilateral paw strikes.	
Rel Step length	%	Step length/stride length*100	
Rel Step time	%	Step time/stride time*100	
Stance time	s	Time between ipsilateral paw strike and successive paw off.	
Swing time	s	Time between ipsilateral paw off and successive paw strike.	
Single support phase	%	Time from contralateral paw off to successive contralateral paw strike/stride time*100	Single support phase + double support phase + swing phase = 100% stride
Double support phase	%	(Time from ipsilateral paw strike to contralateral paw off + time from contralateral paw strike to ipsilateral paw off)/stride time*100	Single support phase + double support phase + swing phase = 100% stride
Swing phase	%	Time between ipsilateral paw off and successive paw strike/stride time*100	Single support phase + double support phase + swing phase = 100% stride
**Kinetic**			
PFz	%	Maximum (peak) vertical ground reaction force during one stride in relation to body weight.	

MFz	%	Mean vertical ground reaction force during one stride in relation to body weight.	
IFz	New*s/ %	Impulse of vertical ground reaction forces during one stride in relation to body weight. (MFz*stance time)	
Range Fz	New	Mean range of vertical ground reaction forces during one stride.	
STDEV Fz	New	Standard deviation of vertical ground reaction forces during one stride.	
Relation PFz T/P	-	Distribution of vertical ground reaction force comparing thoracic to pelvic limbs. (PFz thoracic limb/PFz pelvic limb)	Common: 60/40 to 70/30 = 1,5 to 2,3
Symmetry index PFz	%	SI = 100% - [(PFz left paw /PFz right paw)*100] (modified formula by Budsberg et al. 1993)	>6/<-6 = orthopedic lameness
PFy	%	Maximum (peak) craniocaudal ground reaction force during one stride in relation to body weight.	
Range Fy	New	Mean range of craniocaudal ground reaction forces during one stride.	
STDEV Fy	New	Standard deviation of craniocaudal ground reaction forces during one stride.	
PFx	%	Maximum (peak) mediolateral ground reaction force during one stride in relation to body weight.	
Range Fx	New	Mean range of mediolateral ground reaction forces during one stride.	
STDEV Fx	New	Standard deviation of mediolateral ground reaction forces during one stride.	

Supplementary Table 2. Descriptive statistical analysis of parameter values and coefficients of variation (* p <= 0.05, Mann-Whitney test, FDR Q = 0.05) of spatio-temporal gait parameters. 50 strides of a non-ataxic control group as well as 50 strides of an ataxic study group were analyzed and compared. T = thoracic limbs; P = pelvic limbs; N = number of steps; SD = standard deviation; CV = Coefficient of variation; highlighted cells = absolute values (restricted comparability); See **Supplementary table 1** for detailed definitions of spatio-temporal gait parameters.

**SPATIO-TEMPORAL**	**Control group** (n=6, N=600)				**Study group** (n=5, N=500)			
	**Median**	**Range**	**Variation**	**CV**	**Median**	**Range**	**Variation**	**CV**
**Stride length T**	447.7	353.0-546.9	1756.71	9.40	596.5	153.0-824.1	21339.98	26.92
**Stride length P**	480.4	351.1-572.4	1785.78	8.84	640.4	281.7-817.5	18193.76	22.99
**Step length T**	243.3	186.0-310.1	544.27	9.56	324.9	85.8-466.9	7700.42	28.73
**Step length P**	245.9	168.2-322.4	820.88	11.61	329.5	99.5-495.6	6841.52	26.54
**Stride time T**	0.76	0.57-1.05	<0.01	9.57	1.15	0.44-1.44	0.06	24.07
**Stride time P**	0,76	0.61-1.04	<0.01	9.39	1,15	0.57-1.43	0.05	21.21
**Step time T**	0.38	0.27-0.63	<0.01	11.12	0.57	0.21-0.77	0.02	25.16
**Step time P**	0.38	0.29-0.63	<0.01	10.69	0.57	0.26-0.75	0.01	22.47
**Stance time T**	0.51	0.27-0.91	<0.01	11.73	0.85	0.22-1.05	0.04	26.23
**Stance time P**	0.47	0.34-0.91	<0.01	10.94	0.81	0.31-1.01	0.03	22.95
**Swing time T**	0.25	0.17-0.54	<0.01	12.86	0.29	0.14-0.48	<0.01	23.15
**Swing time P**	0.29	0.16-0.43	<0.01	13.21	0.34	0.15-0.64	0.01	25.44
**rel. Step length T**	54.8	45.2-65.8	10.93	6.03	55.9	27.7-87.2	39.93	11.23
**rel. Step length P**	51.5	39.4-79.5	16.07	7.76	52.9	29.0-76.5	48.52	13.15
**rel. Step time T**	50.0	40.0-62.4	7.89	5.62	50.0	36.2-60.6	12.76	7.15
**rel. Step time P**	50.0	39.4-64.3	6.11	4.94	50.0	35.0-61.5	14.01	7.48

**Single support T**	33.3	23.1-51.4	9.19	9.05	27.1	15.3-47.8	18.17	15.48
**Single support P**	38.9	23.2-45.8	10.31	8.35	31.2	14.4-52.5	25.25	16.10
**Double support T**	33.3	6.7-44.4	18.68	13.09	46.0	13.6-58.5	51.05	15.90
**Double support P**	22.2	14.5-41.8	18.71	18.76	37.3	15.3-57.8	53.61	19.47
**Swing phase T**	33.7	24.1-53.5	9.99	9.44	27.0	17.0-50.0	18.54	15.64
**Swing phase P**	38.9	25.0-45.1	9.81	8.14	31.1	15.6-51.8	24.05	15.72

Supplementary Table 3. Descriptive statistical analysis of parameter values and coefficients of variation (* p <= 0.05, Mann-Whitney test, FDR Q = 0.05) and kinetic gait parameters. 50 strides of a non-ataxic control group as well as 50 strides of an ataxic study group were analyzed and compared. T = thoracic limbs; P = pelvic limbs; N = number of steps; SD = standard deviation; CV = Coefficient of variation; highlighted cells = absolute values (restricted comparability); See **Supplementary table 1** for detailed definitions of kinetic gait parameters.

**KINETIC**	**Control group** (n=6, N=600)				**Study group** (n=5, N=500)			
	**Median**	**Range**	**Variation**	**CV**	**Median**	**Range**	**Variation**	**CV**
**PFz T**	59.4	45.7-72.6	12.79	6.02	56.2	15.4-83.6	30.81	9.97
**PFz P**	35.2	26.1-51.6	18.08	11.92	33.9	9.5-59.9	30.39	16.15
**MFz T**	40.9	28.4-48.0	7.60	6.79	38.2	8.8-51.9	19.03	11.54
**MFz P**	25.1	15.8-33.7	3.95	7.87	23.8	2.8-34.7	14.32	15.89
**PFy T**	13.2	6.0-30.8	8.99	22.14	10.1	3.2-20.9	10.61	30.96
**PFy P**	5.9	0.0-16.5	13.44	61.18	4.3	0.0-15.6	6.40	53.60
**PFx T**	6.6	0.0-28.4	28.56	79.71	3.9	0.0-23.7	17.86	87.46
**PFx P**	6.4	0.0-16.8	26.45	79.24	3.1	0.0-16.7	13.86	94.84
**IFz T**	20.7	9.8-36.4	7.48	13.16	31.0	4.3-44.5	60.02	26.79
**IFz P**	11.9	6.3-22.1	2.52	13.30	18.4	1.9-25.14	15.44	22.74
**Range Fz T**	86.4	58.5-119.0	101.58	11.59	169.0	31.9-266.2	4691.49	42.77
**Range Fz P**	51.9	22.3-84.6	81.04	17.30	113.7	21.5-160.6	1483.45	40.24
**STDEV Fz T**	23.3	17.2-35.1	9.82	13.24	50.1	9.1-83.5	446.86	45.74
**STDEV Fz P**	11.3	5.2-21.5	6.85	22.34	29.9	4.7-49.8	125.03	44.53
**Range Fy T**	27.1	12.3-57.9	28.72	19.56	46.7	7.8-85.3	427.18	44.58
**Range Fy P**	23.0	11.0-47.8	42.12	26.98	37.7	7.2-79.6	219.97	40.66
**STDEV Fy T**	23.0	11.0-47.8	2.18	18.28	37.7	7.2-79.6	41.66	46.76
**STDEV Fy P**	5.8	3.2-11.0	2.22	24.46	9.0	1.6-18.4	14.45	44.08
**Range Fx T**	19.0	11.0-46.6	11.78	17.67	25.8	7.4-73.6	77.43	33.35
**Range Fx P**	17.6	7.2-39.3	47.38	37.33	20.5	6.0-55.1	57.80	36.62
**STDEV Fx T**	4.9	3.0-13.4	1.30	21.91	6.0	1.7-14.0	3.64	30.42
**STDEV Fx P**	3.8	1.7-9.5	4.03	45.33	4.8	1.4-9.9	2.02	30.57

Supplementary Table 4. Statistical analysis of symmetry indices (* p <= 0.05, Mann-Whitney test, FDR Q = 0.05). 50 strides of a non-ataxic control group as well as 50 strides of an ataxic study group were analyzed and compared. T = thoracic limbs; P = pelvic limbs; See **Supplementary table 1** for detailed definitions.

**SYMMETRY INDICES**	**Parameter values**	
	**P value**	**FDR threshold**
**Symmetry Index T**	0.9307	0.05
**Symmetry Index P**	>0.9999	0.05
